# Identification of a unique endoplasmic retention motif in the *Xenopus* GIRK5 channel and its contribution to oocyte maturation

**DOI:** 10.1002/2211-5463.13113

**Published:** 2021-03-03

**Authors:** Claudia I. Rangel‐Garcia, Carolina Salvador, Karla Chavez‐Garcia, Beatriz Diaz‐Bello, Zinaeli Lopez‐Gonzalez, Lourdes Vazquez‐Cruz, Julio Angel Vazquez‐Martinez, Vianney Ortiz‐Navarrete, Hector Riveros‐Rosas, Laura I. Escobar

**Affiliations:** ^1^ Departamento de Fisiologia Facultad de Medicina Universidad Nacional Autonoma de Mexico Ciudad de Mexico Mexico; ^2^ Departamento de Biomedicina Molecular Centro de Investigacion y de Estudios Avanzados del Instituto Politecnico Nacional Mexico City Mexico; ^3^ Departamento de Bioquimica Facultad de Medicina Universidad Nacional Autonoma de Mexico Ciudad de Mexico Mexico

**Keywords:** endoplasmic retention motif, G protein‐activated potassium channel, nested genes, oocyte, *Xenopus*

## Abstract

G protein‐activated inward‐rectifying potassium (K^+^) channels (Kir3/GIRK) participate in cell excitability. The GIRK5 channel is present in *Xenopus laevis* oocytes. In an attempt to investigate the physiological role of GIRK5, we identified a noncanonical di‐arginine endoplasmic reticulum (ER) retention motif (KRXY). This retention motif is located at the N‐terminal region of GIRK5, coded by two small exons found only in *X. laevis* and *X. tropicalis*. These novel exons are expressed through use of an alternative transcription start site. Mutations in the sequence KRXY produced functional channels and induced progesterone‐independent oocyte meiotic progression. The chimeric proteins enhanced green fluorescent protein (EGFP)‐GIRK5‐WT and the EGFP‐GIRK5K13AR14A double mutant, were localized to the ER and the plasma membrane of the vegetal pole of the oocyte, respectively. Silencing of GIRK5 or blocking of this channel by external barium prevented progesterone‐induced meiotic progression. The endogenous level of GIRK5 protein decreased through oocyte stages in prophase I augmenting by progesterone. In conclusion, we have identified a unique mechanism by which the expression pattern of a K^+^ channel evolved to control *Xenopus* oocyte maturation.

## Graphical abstract

The *Xenopus* GIRK5 potassium channel is orthologous to human GIRK4. The phosphorylatable motif KRXY at the N‐terminus retains GIRK5 at the endoplasmic reticulum. This retention motif is contained in one of two small exons found only in X. *laevis* and X. *tropicalis*. Any mutation or dephosphorylation of tyrosine in KRXY allows delivery of GIRK5 to the plasma membrane at the vegetal pole, promoting oocyte maturation. This unique *Xenopus* retention motif was acquired late in evolution.

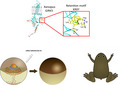

**Abbreviations**
AC: adenylyl cyclase
ECFP: enhanced cyan fluorescent protein
EGFP: enhanced green fluorescent protein
ER: endoplasmic reticulum
GIRK: G protein‐activated inward‐rectifying potassium channels
GVBD: germinal vesicle breakdown
Kir: inwardly rectifying potassium channels

Potassium (K^+^) channels play a crucial role in the life cycle of several organisms by modulating ion homeostasis, cell signaling, cell cycle, and cell death.

Inwardly rectifying potassium channels (Kir) play pivotal roles in controlling insulin release, vascular tone, heart rate, neuronal signaling, and membrane excitability. Among the broad variety of reported K^+^ channels, Kir channels comprise a superfamily found in a wide variety of cells [1], regulating a myriad of metabolic and/or physiological processes [2]. From a functional point of view, Kir channels can be classified in four groups: (a) constitutively active Kir channels (classical Kir2.X channels), (b) G protein‐coupled inwardly rectifying potassium channels (Kir3.X or GIRK channels regulated by G protein‐coupled receptors), (c) ATP‐sensitive K^+^ channels (Kir6.X channels tightly linked to cellular metabolism), and (d) K^+^ transport channels (Kir1.x, Kir4.x, Kir5.x, and Kir7.x) [1]. GIRK channels also regulate cell development and differentiation in lung [3] and breast cancer [4, 5, 6] and mediate vasodilation in smooth muscle cells [7].

GIRK channels form homo or heterotetramers and constitute the native muscarinic K^+^ channel (KACh) activated by the dimer Gβγ. Four members of the Kir3 subfamily, Kir3.1‐Kir3.4 (GIRK1‐GIRK4), have been found in mammals as well as several splice variants [8, 9]. Neurons express different GIRK2 splice variants and GIRK4 is present in the cardiac atrium. Only coassembly of GIRK1 with other GIRK channels produces functional channels [8]. GIRK1 and GIRK4 comprise the KACh that regulates the heart rate [10], while GIRK1/GIRK2 heteromultimer causes slow inhibition of central neurons in the brain due to the action of different neurotransmitters coupled to Gi metabotropic receptors [11]. Whereas GIRK heteromultimer channels often exhibit similar electrophysiological properties, each subtype displays distinct trafficking motifs that control its export from the endoplasmic reticulum (ER) to plasma membrane via endosomes, internalization from the surface, and/or targeting to lysosomes. For example, GIRK1 and GIRK3 homotetramers reside exclusively in the ER; in contrast, GIRK2 and GIRK4 are directed to the cell surface [12].

Another member of this family is GIRK5 from *Xenopus laevis* [13]. Surface expression of GIRK5 displays a constitutive activity due to the endogenous Gβγ pool [14]. Among their mammalian homologues, GIRK5 contains a long N‐terminal sequence (Fig. 1); however, few studies have been performed about the intracellular trafficking of GIRK5 [15].

**Fig. 1:**
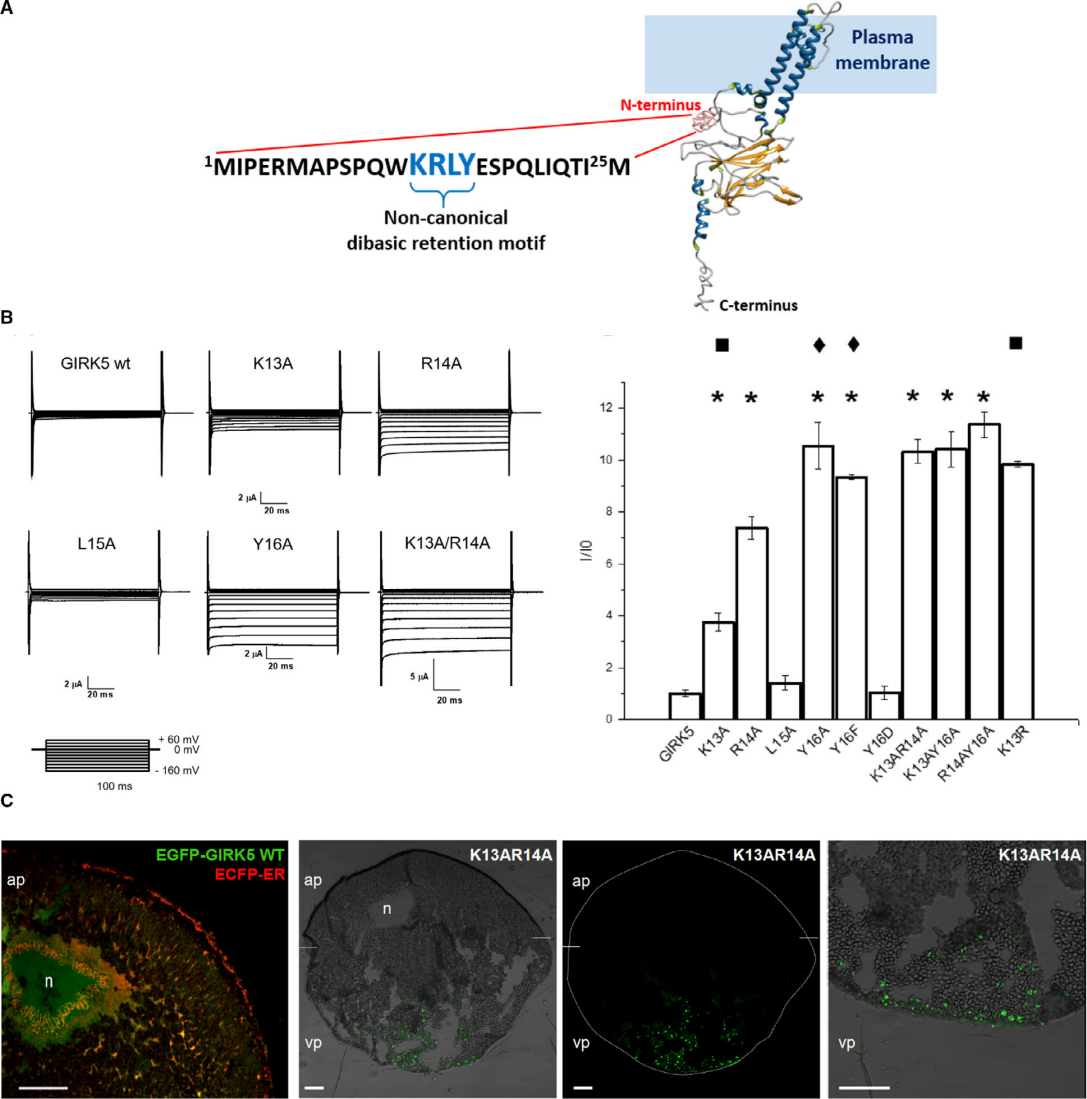
Identification of a noncanonical dibasic retention motif by expression of GIRK5 mutants in *X. laevis* oocytes. (A) The lysine‐arginine‐tyrosine (KRXY) based endoplasmic retention motif at the N terminus of GIRK5 is depicted. 3D structure of GIRK5 was modeled at I‐Tasser server ([67]; https://zhanglab.ccmb.med.umich.edu/I‐TASSER/). The predicted structure was visualized through the UCSF Chimera ([68]; http://www.rbvi.ucsf.edu/chimera/) and secondary structure elements were assigned according to SCOT [69]. (B) Representative current‐time recordings for GIRK5 WT and K13, R14, L15, Y16, K13R14 alanine mutants. Normalized currents (*I*/*I*_0_) were registered at −160 mV. Mutants have a significant difference compared to GIRK5 WT (*, one‐way ANOVA, *P* < 0.01). Based on endogenous phosphorylation of Y16 [30], the effect of a negative charge at Y16 was evaluated with Y16D. There was not a significant difference between Y16A and Y16F (◆) (one‐way ANOVA, *P* < 0.01). Arginine (R) mutant at 13 position did not preserve the retention motif KRXYPi (■). Error bars correspond to mean ± SEM of independent experimental observations (*n* = 12–15) of independent experimental observations. Recording solution contained: 118 mm KCl, 1 mm CaCl_2_, 2 mm MgCl_2_, and 5 mm HEPES, pH 7.4. Recording pipette was filled with 3 m KCl. Voltage pulse protocols were performed using consecutive 100‐ms step changes from −160 to +60 mV with increments of 20 mV. Oocytes were clamped at a holding potential of 0 mV. (C) Confocal and light transmission images of *X. laevis* oocyte sections. The EGFP‐GIRK5 WT and ECFP‐ER constructs were colocalized in the nucleus (*n*) and ER (yellow) of the animal pole (ap); they are shown in the first panel. Merge images of the oocyte injected with the GIRK5K13AR14A‐EGFP construct are shown in the other panels. GIRK5K13AR14A‐EGFP was located in the cytoplasm (vesicles) and plasma membrane of the vegetal pole (vp). Circumference oocyte (in confocal image) and limit between the animal and vegetal poles are indicated on with a dotted line. Scale bar, 100 µm.

The *X. laevis* oocyte has been widely used as a model of maturation and fertilization events. The immature oocyte remains arrested in the late G2 phase of meiosis I [16]. Resumption of meiosis I is produced by the action of progesterone at the oocyte plasma membrane. MOS is one of the first ‘*de novo*’ synthesized proteins [16] that activates the signal transduction: MEK1 (MAPKK), Xp42 MAPK (ERK2), phosphatase Cdc25, Cdc2 and Cyclin B/Cdc2 complex (maturation promoting factor) to resume meiosis [17]. During this process, the germinal vesicle breakdown (GVBD) appears as a white spot at the animal pole of the oocyte and constitutes a morphological marker of meiotic progression.

Changes in membrane potential play an important role in excitable cells and in carcinogenesis in nonexcitable cells [18]. Usually, high negative values (−70 to −90 mV) of the resting membrane potential are displayed in terminally differentiated cells like neurons and muscle cells; in contrast, more depolarized values are found in stem, embryonic, and cancer cells [18]. The resting membrane potential of frog oocytes ranges from −30 to −60 mV in standard saline solution [19].

Some ion channels show variation on their expression or activity across stages of the cell cycle. For example, arrest of proliferating astrocytes in G1/G0 by all‐trans‐retinoic acid induces premature expression of inwardly rectifying K^+^ currents typically expressed in differentiated astrocytes [20]. Also, MCF‐7 cells arrested in G0/G1 have a depolarized resting membrane potential (RMP, −26.3 ± 10 mV) and small outward K^+^ currents density (IK density = 9.4 ± 5.6 pA/pF) compared to cells in the G1 phase (RMP = −60 ± 7.9 mV; IK density = 30.2 ± 8.5 pA/pF) [21].

In the oocyte of *X. laevis*, sodium voltage‐dependent channel activity has been recorded [22] and related to massive efflux of chloride during fertilization, causing instant depolarization of the egg membrane, preventing polyspermy [23]. External neurotransmitters like acetylcholine causes depolarization of *X. laevis* oocytes through an endogenous muscarinic receptor coupled to chloride channels [24]. However, the capacity of oocytes to respond to acetylcholine disappears during meiotic maturation. Furthermore, expression of angiotensin II receptors (AT2) in *X. laevis* oocytes surrounded by follicular cells induces the GVBD process, suggesting that AT2 provokes oocyte maturation by mobilizing intracellular calcium and/or by increasing cGMP [25].

Amphibian immature oocytes display several types of chloride currents that might be related to the establishment of cell polarity during oogenesis [26]. The coordinated action of chloride and sodium channels appears to modulate oocyte maturation in *Rana pipiens*. Chloride channels disappear throughout maturation; in contrast, sodium channels remain active up to full maturity of the oocyte [22].

K^+^ is the main cation inside the cells, determining and regulating the value of their resting membrane potential. Even when K^+^ channels control the timing mechanisms linked to cell cycle in mouse embryos, their molecular identity has remained uncertain [27].

Recording of endogenous inward‐rectifying K^+^ currents in native oocytes of *X. laevis* depends on frog donors and season [28, 29]. Our group has characterized and related this endogenous inwardly rectifying K^+^ current to GIRK5 channel [13]; however, no evidence exists on the role of these K^+^ currents in the oocyte.

Previously, we observed that recombinant GIRK5 was retained in the ER of *X. laevis* oocytes [15]; this retention was linked to phosphorylation of a tyrosine (Y16) at the N terminus [30]. Interestingly, this tyrosine is within the basic sequence KRLY. In animal cells, membrane proteins located in the ER usually carry a di‐lysine motif—KXKXX—at their C‐terminal [31], while others contain a di‐arginine (RR) motif retention/retrieval signal. The RR motif is made of either two consecutive R residues (RRXX, XRRX, XXRR) or R residues separated by an amino acid (RXRX and XRXR) at the N‐terminal region [32, 33]. Furthermore, the arginine–lysine (RK) motif functions only when the two basic residues are placed adjacent to each other (RK, KR) [32].

To investigate the physiological role of GIRK5 in *X. laevis* oocytes, we performed several assays: amino acid scanning mutagenesis of the sequence KRLY16, electrophysiological recordings, channel localization by confocal microscopy, protein silencing by siRNA, progesterone incubation, external barium block, production of a specific antibody, and the identification of GIRK5 orthologs in other species. Through these approaches, we identified a non canonical ER retention motif by which the activity of a GIRK channel evolves in *X. laevis* and *X. tropicalis*. Strikingly, surface expression of GIRK5 promoted progesterone‐independent oocyte maturation. Progesterone‐induced oocyte maturation was prevented by GIRK5 silencing or external barium block of the channel. Through production of a specific antibody, we detected abundant endogenous GIRK5 expression that diminished during oogenesis but increased again by progesterone‐induced maturation.

## Materials and methods

### Sequence analysis

Amino acid sequences belonging to Kir family were retrieved using the BlastP command of DIAMOND [34] against the complete genomes available at NCBI's RefSeq genome database ([35]; ftp://ftp.ncbi.nlm.nih.gov/genomes/refseq) as of September 2020. Progressive multiple amino acid sequence alignments were performed with ClustalX version 2 ([36]; http://www.clustal.org/clustal2/), using as a guide a structural alignment constructed with the VAST algorithm ([37]; http://www.ncbi.nlm.nih.gov/Structure/VAST/vast.shtml). The guiding alignment included all nonredundant Kir protein structures deposited in the Protein Data Bank ([38]; http://www.rcsb.org/) as of September 2020. The sequence alignments obtained were corrected manually using bioedit ([39]; http://en.bio‐soft.net/format/BioEdit.html).

Phylogenetic analyses were conducted using the mega7 software suite ([40]; http://www.megasoftware.net). The evolutionary history was inferred by using the Maximum Likelihood method based on the Jones‐Taylor‐Thornton model. A discrete Gamma distribution was used to model evolutionary rate differences among sites (five categories (+G, parameter = 0.9010)). The rate variation model allowed for some sites to be evolutionarily invariable ([+*I*], 1.66% sites). The tree is drawn to scale, with branch lengths measured in the number of substitutions per site. Confidence for the internal branches of the phylogenetic tree was determined through bootstrap analysis (500 replicates each).

### DNA constructs

Mutants were generated by the two‐step PCR method [15] with GIRK5 cDNA (GenBank ID: NP_001156864.1) as template. Primer SP6 (sense) and Low 2 (antisense) were used as flanking primers (Table S1). To silence GIRK5, double‐stranded siRNA 21‐mer oligonucleotides corresponding to bases 1267–1284 of GIRK5 were designed (Dharmacon, Inc., Waterbeach, Cambridge, UK; Table S1).

Constructs encoding enhanced green fluorescent protein (EGFP) fusion proteins were generated by adding the ORF of pEGFPC1 (Clontech, Palo Alto, CA, USA), to the N terminus of GIRK5 cDNA. The GIRK5 cDNAs and EGFP chimera constructs were subcloned into the pRSSP6013A3‐UWE vector (pBF) and sequenced. The ER‐enhanced cyan fluorescent protein ECFP‐ER cDNA to identify the ER [15] was donated by Luis Vaca (Inst. Fisiol. Cel., UNAM, CDMX, Mexico).

### mRNA synthesis and microinjection

*Xenopus laevis* frogs were anesthetized with tricaine 0.2%. Ovarian lobes were digested with 2 mg·mL^−1^ type IA collagenase (Merck‐Sigma‐Aldrich Quimica, S.L. Toluca, Estado de Mexico, Mexico)

in a Ca^2+^‐free ND96 solution during 50 min to remove follicle cells. Animal experiments were approved by the Committee on Animal Research and Ethics at the Facultad de Medicina, under the official Mexican standard NOM‐062‐ZOO‐1999 (approval number 11‐2017). Oocytes were maintained in ND96 solution (96 mm NaCl, 2 mm KCl, 1.8 mm CaCl_2_, 1 mm MgCl_2_, 5 mm HEPES, 2.5 mm sodium pyruvate; pH 7.4) at 18 °C. mRNAs of all GIRK5 mutants were synthesized and injected in mature oocytes (stage VI) as described previously [15]. Ten nanogram of double‐stranded GIRK5 siRNA 21‐mer was injected per oocyte. An equal amount of scrambled siRNA was injected as a control.

### Electrophysiology

Whole‐cell currents were recorded 48–72 h after mRNA injection with a two‐electrode voltage clamp amplifier (Geneclamp 500) and a Digidata 1322A interface with pClamp8 software (Axon Instruments, San Jose, CA, USA) at room temperature (22–24 °C), as previously described [30]. The recording solution contained: 118 mm KCl, 1 mm CaCl_2_, 2 mm MgCl_2_, and 5 mm HEPES, pH 7.4. The recording pipette was filled with 3 m KCl. Voltage pulse protocols were performed using consecutive 100 ms step changes from −160 to +60 mV with increments of 20 mV. Oocytes were clamped at a holding potential of 0 mV. Data were sampled at 5–10 kHz and filtered at 1–5 kHz. Error bars correspond to the mean ± SEM from a number (*n*) of independent experimental observations. Analysis of variance (One‐Way ANOVA) test was applied to compare the significant differences between the experiments (*P* < 0.01). origin v. 8.1 (OriginLab, Northampton, MA, USA) and prism v.1 (GraphPad Software, San Diego, CA, USA) were used.

### Confocal microscopy

Forty‐eight hours after mRNA injection oocytes was fixed with 2% paraformaldehyde (PFA; Merck‐Sigma‐Aldrich Quimica, S.L. Toluca Estado de Mexico, Mexico) in ND96 for 10 min. Oocytes were incubated in 30% sucrose, 2% PFA, ND96, and kept at 4 °C. Serial 10‐μm slices were cryostat‐cut (Leica CM1100, CDMX, Mexico) at −20 °C, mounted on gelatin‐coated slides, and cover‐slipped with Vectashield mounting medium (Vector Laboratories, Burlingame, CA, USA). Images were obtained with the Nikon A1R HD25 confocal microscope using a 10× objective. Images were analyzed with NIS Elements Advanced Research software (Nikon, Melville, NY, USA). EGFP fluorescence was excited with an argon laser beam at 488 and emitted fluorescent light was collected between 496 and 530 nm. ECFP fluorescence was excited at 458 nm and fluorescent emission was collected in blue between 460 and 495 nm and changed to red to facilitate the merge. Photographs were taken at the midsection of each oocyte.

### Maturation assays

Oocyte maturation was stimulated with progesterone and validated by pERK2/ERK2 immunoblot analysis and GVBD counting. Batches of 40 defolliculated oocytes (stage VI) noninjected and injected with GIRK5K13AR14A and/or GIRK5 siRNA were incubated in 2 mL of ND96 with 10 μm progesterone for 1, 3, 5, 7, and 10 h according to Serrano‐Flores [41]. To study the effect of barium on GVBD (Fig. 5), non injected and injected oocytes with the KR double‐alanine mutant were incubated with barium (111 μm) during the progesterone treatment.

### Anti‐GIRK5 production

B‐Cell Epitope Predictor algorithm, BepiPred‐2.0, of IEDB Analysis Resource (http://tools.iedb.org/bcell/) was used to analyze the sequence of GIRK5 protein (GenBank ID: NP_001156864.1). The predicted epitope (GIRK5_5‐22_) was synthesized as Multi‐Antigenic Peptide (MAP4) by Biosynthesis (Lewisville, TX, USA). Four‐week‐old rabbits were immunized with 500 µg·mL^−1^ of MAP4‐GIRK5_5‐22_ emulsified in Freund's complete adjuvant, followed by two immunizations in Freund's incomplete adjuvant, and peptide in PBS in the subsequent three immunizations. IgG was purified from the antisera by HiTrap Protein G HP (GE Healthcare, 17040501, Merck‐Sigma‐Aldrich Quimica) according to the manufacturing protocol. All animal experiments were performed according to the Guide for Laboratory Animals and Official Mexican Norm (NOM‐062‐ZOO‐1999) and approved by the Cinvestav Institutional Committee for Care and Use of Laboratory Animals (approval number 0078‐14).

### Immunoblots

Oocytes were lysed by pipetting up and down 20 times in 180 μL of ice‐cold extraction buffer with lysis buffer (250 mm sucrose, 1 mm EDTA, 1 mm Tris/HCl, pH 7.6) and protease inhibitors cocktail (cOmplete Mini Roche, Merck‐Sigma‐Aldrich Quimica). The samples were centrifuged two times at 200 ***g*** and once at 14 000 ***g*** for 5 min at 4 °C. For EGFP‐GIRK5 channel immunoblotting, the homogenate was centrifuged three times at 1000 ***g*** for 10 min. The supernatant was collected and centrifuged for 1 h at 150 000 ***g***. The pellet (membrane fraction) was dissolved in 200 µL of lysis buffer and centrifuged at 150 000 ***g*** for 20 min. The pellet was suspended in 30 μL of lysis buffer. Protein concentration was determined with DC protein assay (Bio‐Rad Laboratories CDMX, Mexico). Samples (30 μg of protein) were denatured in 2 μL of Laemmli buffer per oocyte with 5% β‐mercaptoethanol at 95 °C for 10 min, rinsed in 20 μL of sample buffer and separated by 10 % SDS/PAGE and electro‐blotted to a PVDF membrane, which was blocked with 5% nonfat dry milk (Bio‐Rad, Crissof, CDMX, Mexico) in TBS‐T (Tris‐buffered saline pH 7.6, 1% Tween‐20) for 1 h at room temperature.

For immunodetection of the GIRK5‐K13AR14A EGFP construct, a monoclonal antibody against EGFP was used (1 : 1000 dilution; Clontech). The secondary antibody was a horseradish peroxidase‐conjugated goat anti‐mouse IgG (1 : 10 000 dilution; Amersham Life Science, Merck‐Sigma‐Aldrich Quimica).

For maturation assays, the membranes were incubated overnight at 4 °C with anti‐pERK2 monoclonal antibody (1 : 200 dilution; SC‐136521; Santa Cruz Biotechnology, Dallas, TX, USA). Anti‐mouse IgG secondary antibody (1 : 5000 dilution; SC‐2314; Santa Cruz) was used. Antibodies were removed by stripping procedure (100 mm β‐mercaptoethanol, 2% sodium dodecyl sulfate, 62.5 mm Tris/HCl, pH 6.7 buffer at 60 °C for 30 min) and the membranes were re‐incubated with anti‐ERK2 monoclonal antibody, used as loading control (1 : 2500 dilution; SC‐1647; Santa Cruz). Signal intensity of pERK2 bands was normalized to ERK2. For endogenous GIRK5 expression assays, the membrane was incubated with anti‐GIRK5 polyclonal antibody overnight at 4 °C (dilution 1 : 3000). Anti‐rabbit IgG secondary antibody was used (1 : 5000 dilution; 7074; Cell Signaling Technology, Danvers, MA, USA). Immunoreactivity was detected using Immobilon Western Chemiluminescent HRP Substrate (Millipore, Burlington, MA, USA) and autoradiography films (Kodak, Merck‐Sigma‐Aldrich Química, S.L. Toluca, Estado de Mexico, Mexico).

To evaluate endogenous GIRK5 expression during oogenesis, oocytes from all stages (I‐VI) were separated under a stereoscopic microscope. Additionally, oocytes were incubated with progesterone (10 μm, 7 h). Each group was processed for immunoblot analysis with our anti‐GIRK5 antibody. For statistical analysis, band density was compared between groups by one‐way ANOVA test (*P* < 0.05).

## Results

### Identification of a noncanonical retention signal

We evaluated the putative dibasic ER motif KRLY located at the N terminus of GIRK5 (Fig. 1A) by introducing single‐ and double‐alanine mutations: K13A, R14A, L15A, Y16A, K13AR14A, K13AY16A, R14AY16A, and mutants Y16F, Y16D and K13R. As expected, current sizes of GIRK5 WT did not show significant differences with noninjected oocytes: 0.89 ± 0.18 µA and 0.653 ± 0.04 µA at −160 mV, respectively (Fig. 1B). Except for L15A and the negatively charged mutant Y16D that probably were retained at the ER as is the case with GIRK5 WT [15], all mutants produced functional channels by reaching the plasma membrane and generating currents of variable magnitude (Fig. 1B).

To compare the localization of GIRK5 and the KR double‐alanine mutant, their EGFP chimeras were produced, injected in oocytes, and observed by confocal microscopy. Previously, we found EGFP‐GIRK5 WT localized to the nucleus, perinuclear space and ER in the animal pole of *X. laevis* oocytes [15]. As expected, K13R14A EGFP construct was not retained in the ER but observed distributed throughout the cytoplasm (most probably in vesicles) and plasma membrane of the vegetal pole (Fig. 1C). These results supported that the KRXY sequence at the NH2‐terminal of GIRK5 works as an ER retention signal. Interestingly, it has been documented that in a di‐arginine retention motif RRXX, a lysine (K) can replace any of the R residues (i.e. KR or RK) [32, 33]. However, when we replaced K13 by R this mutant elicited high inwardly rectifying K^+^ currents. Therefore, position 13–16 of KRXY residues, but not RRXY, at the N terminus, is determinant to maintain GIRK5 at the ER.

### The noncanonical KRLY retention signal of GIRK5 is conserved exclusively in *Xenopus*

**Fig. 2:**
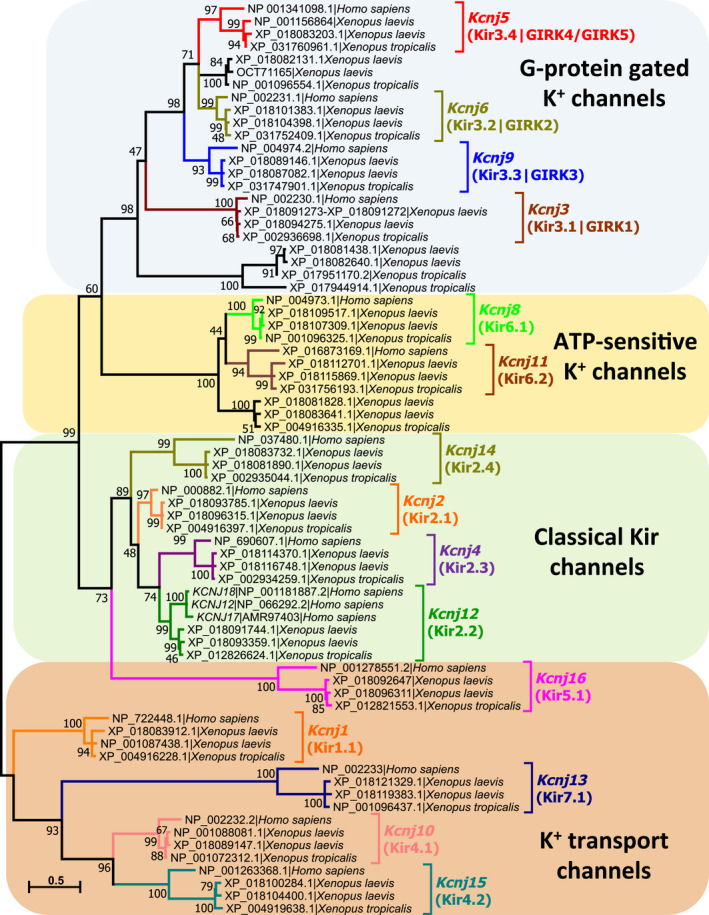
Phylogenetic analysis of inwardly rectifying potassium (Kir) channels present in human, *X. laevis* and *X. tropicalis*. The phylogenetic tree was constructed with 72 Kir protein sequences (17 from human, 19 from *X. tropicalis*, and 36 from *X. laevis* (a tetraploid frog) that can be classified into four functional groups. Accession numbers of each protein sequence as well as Kir channel subfamilies previously described in humans are indicated. The evolutionary history was inferred from 500 replicates, using the Maximum Likelihood method based on the Jones–Taylor–Thornton matrix‐based model w/freq. The tree with the highest log likelihood (−21 352.85) is shown. Evolutionary analyses were conducted in MEGA7 [40], considering a total of 581 positions in the final dataset. Tree branches are drawn to scale, with the bar length indicating the number of substitutions per site. The percentage of trees in which the associated taxa clustered together in the bootstrap test (500 replicates) is shown next to the branches. Branch lengths represent the number of substitutions per site as indicated by the bar.

To determine whether the KRLY retention signal was found in other Kir channels, homologous amino acid sequences were retrieved using DIAMOND BlastP searches against the complete animal genomes available at NCBI’s RefSeq genome database. A total of 7346 protein sequences identified as Kir channels were found in the 561 completely sequenced animal genomes. With exception of Platyhelminthes (*Schistosoma mansoni, S. haematobium, Echinococcus granulosus, and Opisthorchis viverrini*), the rest of animals with a completely sequenced genome possess at least one gene coding for a Kir channel (see Data S1). Humans possess 17 Kir channels encoded by *KCNJ* genes (*KCNJ1* to *KCNJ6*, and *KCNJ8* to *KCNJ18*). In contrast, we found 19 *kcnj* genes in *X. tropicalis* genome and 36 *kcnj* genes in *X. laevis* genome (the higher number of *kcnj* genes in *X. laevis* is due to the fact that *X. laevis* is a tetraploid frog, while *X. tropicalis* is a diploid frog). A phylogenetic tree constructed with the Kir channel amino acid sequences found in humans, *X. tropicalis* and *X. laevis* (Fig. 2), shows that for each human *KCNJ* gene, *X. tropicalis* and *X. laevis* possess one or two ortholog genes, respectively. Only human *KCNJ17* and *KCNJ18* [42] are absent in *Xenopus* genomes. Our phylogenetic analysis (Fig. 2) and *KCNJ12* gene synteny analysis (not shown) strongly suggest that these last two human genes originated from a recent *KCNJ12* gene duplication.

Human possess four GIRK channels (regulated by G protein‐coupled receptors) coded by *KCNJ3* (GIRK1), *KCNJ5* (GIRK4), *KCNJ6* (GIRK2), and *KCNJ9* (GIRK3) genes (Fig. 2 and Table 1). *Xenopus* GIRK5, coded by *Kcnj*5 gene, is orthologous to the human GIRK4. Interestingly, *Xenopus* has two additional putative GIRK channels not present in humans.

**Table 1:** G protein‐gated K^+^ channels that are present in *X. laevis, X. tropicalis,* and humans.

*Gene* (protein name)	*Xenopus laevis*	*Xenopus tropicalis*	*Homo sapiens*
	Protein accession number (sequence length) [chromosomal location]		
*Kcnj6* Kir3.2 GIRK2	XP_018101383 (405 aa) [chromosome 2L] XP_018104398 (405 aa) [chromosome 2S]	XP_031752409 (405 aa) [chromosome 2]	NP_002231 (423 aa) [21q22.13]
*Kcnj5* Kir3.4 (human) Kir3.5 (*Xenopus*) GIRK4 (human) GIRK5 (*Xenopus*)	NP_001156864 (429 aa) [chromosome 7L] XP_018083203 (201 aa) [chromosome 7S]	XP_031760961 (440 aa) [chromosome 7]	NP_001341098 (419 aa) [11q24.3]
*Kcnj9* Kir3.3 GIRK3	XP_018087082^a^ (411 aa) [chromosome 8L] XP_018089146^a^ (309 aa) [chromosome 8S]	XP_031747901^a^ (411 aa) [chromosome 8]	NP_004974 (393 aa) [1q23.2]
*Kcnj3* Kir3.1 GIRK1	XP_018091273‐XP_018091272^b^ (489 aa) [chromosome 9_10L] XP_018094275 (489 aa) [chromosome 9_10S]	XP_002936698 (489 aa) [chromosome 9]	NP_002230 (501 aa) [2q24.1]
Incorrectly reported as *Kcnj9* at NCBI^c^	XP_018082131 (418 aa) [Chromosome 7L] OCT71165 (393 aa) [chromosome 7S]	NP_001096554 (407 aa) [Chromosome 7]	Absent
Not given yet official gene name (Putative G protein‐gated K^+^ channel)	XP_018081438 (430 aa) [chromosome 7L] XP_018082640 (336 aa) [chromosome 7S]	XP_017951170 (603 aa) [chromosome 7] XP_017944914 (486 aa) [chromosome 7]	Absent

^a^ True ortholog of the human *KCNJ9* gene.

^b^ Reported as two gene fragment (both fragments were merged *in silico* to obtain a complete sequence).

^c^ National Center for Biotechnology Information:https://www.ncbi.nlm.nih.gov/gene/.

**Fig. 3:**
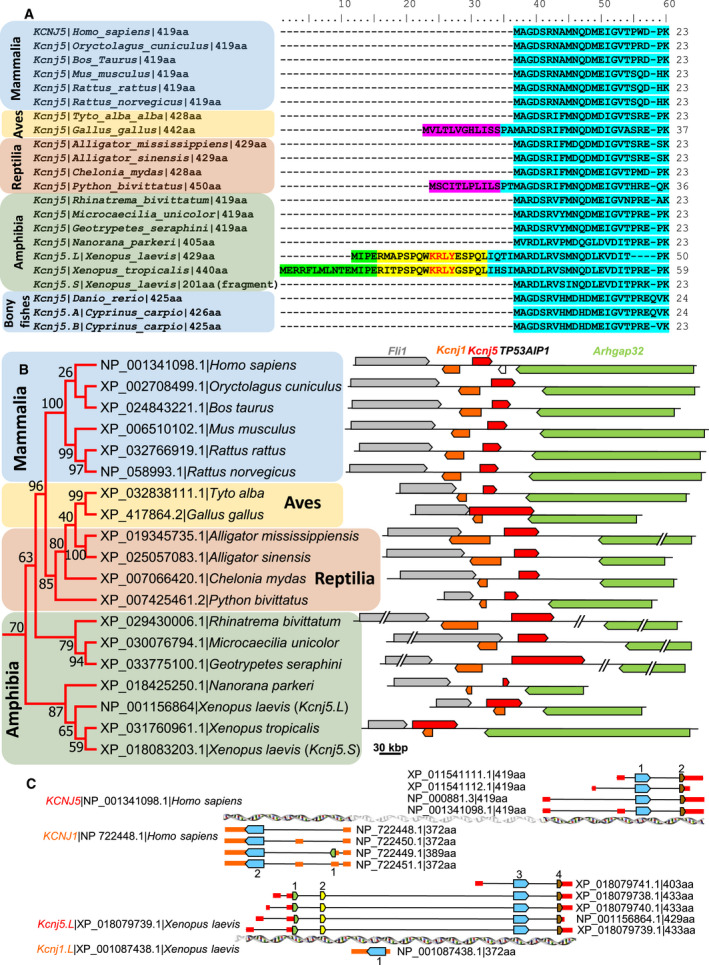
Sequence analysis of some selected GIRK4/GIRK5 channels that belong to fishes, amphibians, reptiles, avian, and mammals. (A) Multiple amino acid sequence alignment of 22 GIRK4/GIRK5 channels showing the N termini of the proteins: residues from homologous exons are lighted with the same color (green, yellow, pink, or light blue). The noncanonical KRLY retention signal of GIRK5 from *X. laevis* and *X. tropicalis* is indicated in red letters into their second exon (yellow lighted). Sequence names indicate coding gene | species name | protein sequence length. (B) Phylogenetic analysis and gene context (synteny) of selected GIRK4/GIRK5 channels. The phylogenetic tree was constructed with 22 GIRK4/GIRK5 protein sequences coded by *Kcnj5* genes. Sequence names indicate accession number for each GIRK4/GIRK5 protein channel followed by the species name. The evolutionary history was inferred from 500 replicates, using the Maximum Likelihood method based on the Jones–Taylor–Thornton matrix‐based model w/freq. Percent bootstrap values are shown next to the branches. Evolutionary analyses were conducted in MEGA7 [40]. The gene context (synteny) of each *Kcnj5* gene is indicated to the right of each sequence included in the tree (arrows represent genes and direction of transcription). Flanking *Kcnj1–Kcnj5* gene pair (coding for Kir1.1 and GIRK4/GIRK5 channels, respectively), *Arhgap32* gene (encoding a Rho GTPase activating protein 32), and *Fli1* (encoding Friend leukemia integration 1 (*Fli‐1*) proto‐oncogene) can be found; in humans, *TP53AIP1* gene, coding for tumor protein p53 regulated apoptosis inducing protein 1 is located between *KCNJ5* and *ARHGAP32* genes. GIRK4/GIRK5 sequences from fishes are not shown because their synteny is not conserved; and synteny of *X. laevis Kcnj5.S* is not shown because the available sequence is just a fragment. Genes are drawn on the same scale. (C) intron–exon structure comparison between *Kcnj1 and Kcnj5* gene pair from human and *X. laevis*. Coding exons are indicated by arrows (colored according to the colors used in panel A), untranslated region by short boxes (colored red for *Kcnj5*, and orange for *Kcnj1*), and introns by black lines.

On the other hand, from 7346 protein sequences identified as Kir channels, 738 were identified as orthologs of *Xenopus* GIRK5. Figure 3A shows the N‐terminal region of a multiple sequence alignment of some selected GIRK5 orthologs that belong to fishes, amphibia, reptiles, avian, and mammals. The initial methionine for the majority of GIRK5/GIRK4 protein sequences corresponds to M26 or M37 from *X. laevis* and *X*. *tropicalis* GIRK5, respectively. This suggests that the first amino acid residues, located in the N terminus of GIRK5 (25 in *X. laevis* and 36 in *X. tropicalis*), were acquired late in evolution. Indeed, a search of the KRLY retention signal in the 7346 protein sequences identified as Kir channels revealed that this retention signal is found exclusively in *Xenopus* GIRK5.

### A noncanonical KRLY retention signal in *Xenopus* GIRK5 emerged by acquisition of two novel exons at the N terminus

Synteny analysis shows that *Kcnj5* gene (encoding GIRK4/GIRK5 or Kir3.4/Kir3.5 channels) is adjacent to *Kcnj1* gene (encoding Kir1.1) in amphibia, reptiles, avian, and mammals (Fig. 3B). Both genes are flanked by *ARHGAP32* gene (encoding a Rho GTPase activating protein 32 and *FLI1* proto‐oncogene 32, encoding Friend leukemia integration 1 (FLI‐1) transcription factor). The size of *Kcnj5* gene is highly variable into different species, and span from 9664 bp in *Nanorana parkeri* (Tibetan frog) to 112 613 bp in *Geotrypetes seraphini* (Gaboon caecilian). Since the size of the amino acid sequence of GIRK4/GIRK5 channels is similar, the very marked variation in *Kcnj5* gene is because introns vary considerably in size. Interestingly, in *Gallus gallus* (chicken), *X. tropicalis,* and *X. laevis,* the *Kcnj1* gene is overlapped or nested within *Kcnj5* gene.

Figure 3C shows an intron–exon structure comparison between *Kcnj1* – *Kcnj5* gene pair from human and *X. laevis*. Human *KCNJ5* gene exhibits three transcription start sites, allowing the synthesis of four possible pre‐mRNAs with three or four exons (Fig. 3C). However, since translation initiates in the common exon, the different mature mRNAs containing different 5′ untranslated regions, encode identical human GIRK4 proteins. Human *KCNJ1* gene exhibits one transcription start site, but four possible pre‐mRNAs with two, three, or four exons. Since translation initiates in two different exons, human *KCNJ1* gene encoded two isoforms containing different N termini.

On the other hand, *X. laevis Kcnj5* gene, located at chromosome 7L (*Kcnj5.L*), has five transcription start sites and five different pre‐mRNAs, each one with three or five exons. Three different GIRK5 isoforms containing different N termini can be encoded by the *Kcnj5* gene. The small isoform of GIRK5 is encoded by a mature mRNA transcript similar to the mature mRNA produced by human *KCNJ5* gene, but the large isoforms of GIRK5 are produced because in addition to the two translated exons observed in humans, two upstream small novel exons located at the N‐term express the first 25 amino acid residues of GIRK5, containing the KRLY retention signal. Interestingly, these novel translated exons of *X. laevis Kcnj5.L* gene are located in chromosome 7L, upstream from *Kcnj1* gene encoded by the opposite DNA strand. Thus, *X*. *laevis Kcnj1* becomes a nested gene after *Kcnj5.L* gene acquires these two additional exons at the N terminus. Since these two novel expressed exons are observed only in *X. laevis Kcnj5.L* and *X. tropicalis Kcnj5*, but not in other tetrapods including other amphibian relatives, we can conclude that these exons containing the KRLY retention motif were acquired recently in the evolutionary history of *Xenopus*.

### Surface expression of GIRK5 by disruption of the KRXY motif accelerates oocyte maturation

**Fig. 4:**
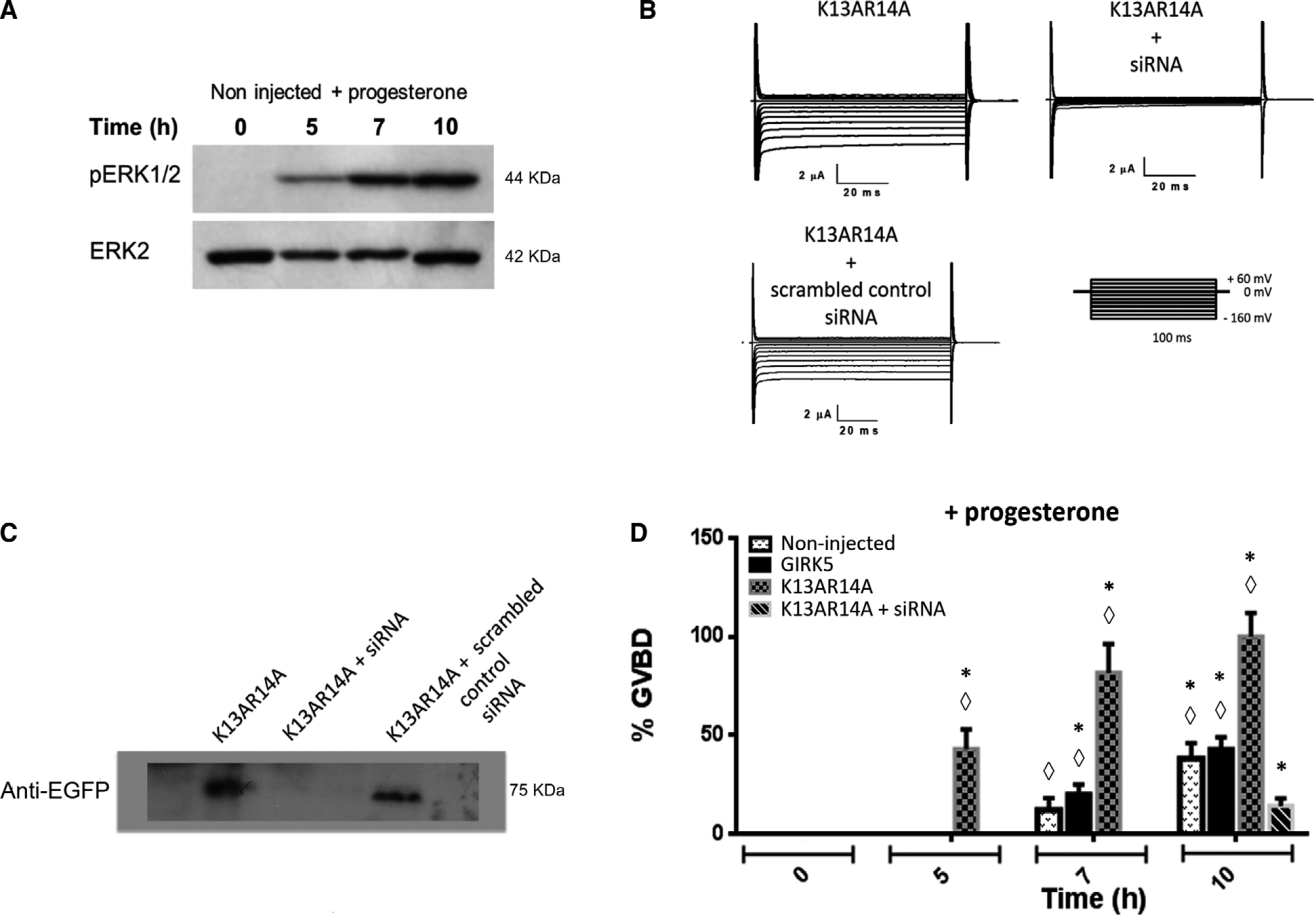
Disruption of the KRXY motif accelerates *X. laevis* oocyte maturation. (A) pERK1/2 (44 kDa) immunoblotting of noninjected oocytes incubated at 5, 7, and 10 h with progesterone (10 μm); ERK2 (42 kDa) was used as a loading control (representative of three independent experiments). (B) Current traces recorded for the GIRK5‐K13AR14A mutant. Voltage pulse protocols were performed using consecutive 100 ms step changes from −160 to +60 mV with increments of 20 mV. Oocytes were clamped at a holding potential of 0 mV. Silencing the gene encoding GIRK5 by specific siRNA suppressed K^+^ currents. (C) GIRK5 silencing was confirmed by EGFP‐GIRK5K13AR14A (75 kDa) immunoblotting (representative of three independent experiments). (D) %GBVD was determined for noninjected and oocytes expressing: GIRK5 WT, GIRK5K13AR14A, and GIRK5K13AR14A plus siRNA GIRK5. Only GIRK5K13AR14A allowed progesterone‐induced oocyte maturation up to 10 h. (*) Significant difference in oocyte maturation expressing WT or GIRK5K13AR14A compared to silenced GIRK5K13AR14A. (◊) Significant difference in the maturation of oocytes expressing GIRK5K13AR14A at 5, 7 and 10 h compared to WT and noninjected oocytes (One‐Way ANOVA*, P* < 0.01). Error bars correspond to mean ± SE, *n* = 15.

Unexpectedly, functional mutants provoked progesterone‐independent oocyte maturation, being mutant K13AR14A the most efficient. Western blot analysis was performed with non‐injected oocytes incubated with progesterone, to determine the level of phosphorylated ERK1/2 (pERK1/2) (Fig. 4A).

Functional expression assays with the double mutant GIRK5K13AR14A demonstrated the success of specific siRNA silencing GIRK5 in oocytes. As expected, oocytes injected with GIRK5K13AR14A cRNA plus scrambled control siRNA elicited inwardly currents (Fig. 4B). EGFP‐GIRK5K13AR14A immunoblotting also confirmed GIRK5 silencing by siRNA (Fig. 4C).

The time‐course of GVBD was followed for non‐injected oocytes, recombinant GIRK5K13AR14A and GIRK5, and GIRK5 silencing in oocytes incubated with progesterone to defy oocyte maturation. K13R14A mutant accelerated oocyte maturation reaching GVBD 40% (5 h), 75% (7 h), and 100% (10 h); in contrast, GIRK5 silencing did not allow progesterone‐induced oocyte maturation (Fig. 4D).

**Fig. 5:**
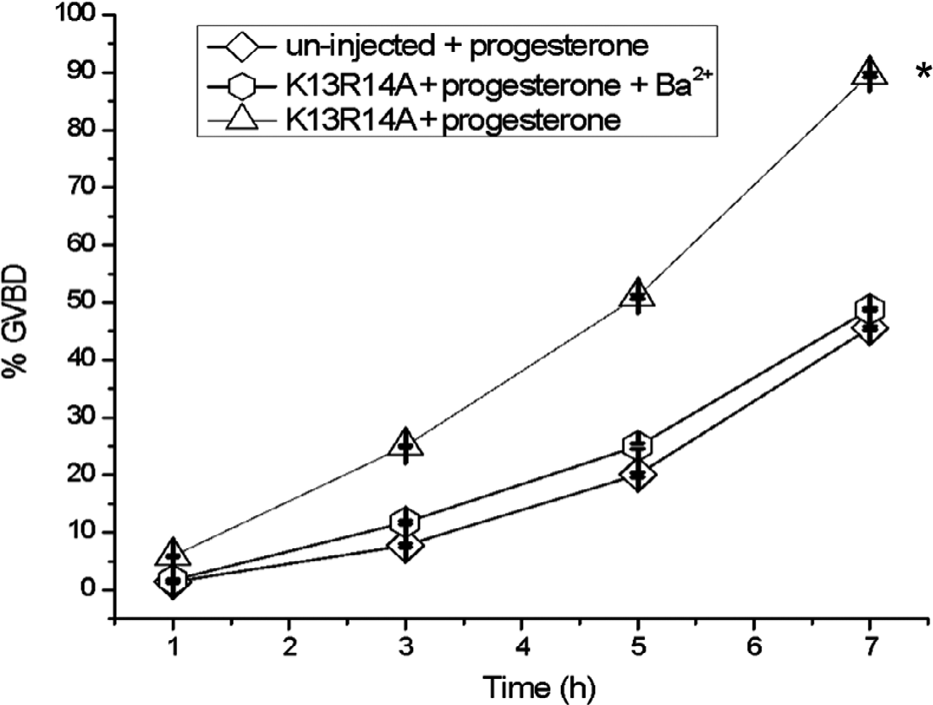
Effect of external barium block on GIRK5‐progesterone‐induced oocyte maturation. %GVBD was determined after 7 h of incubation in the ND96 solution, barium (111 μm) and progesterone (10 μm). (*) indicates a significant difference with respect to the other treatments (one‐way ANOVA*, P* < 0.01). Error bars correspond to mean ± SE, *n* = 15.

In a previous work, we described the prolonged inactivation and voltage‐independent barium blockade of GIRK5 [14]. Therefore, we determined the effect of external barium (111 µm) on progesterone‐induced maturation of oocytes expressing GIRK5K13AR14A. Interestingly, external barium inhibited GVBD (Fig. 5). Therefore, GIRK5 silencing and barium block assays support that GIRK5 channel activity is necessary for oocyte maturation.

### Endogenous GIRK5 expression during oogenesis

**Fig. 6:**
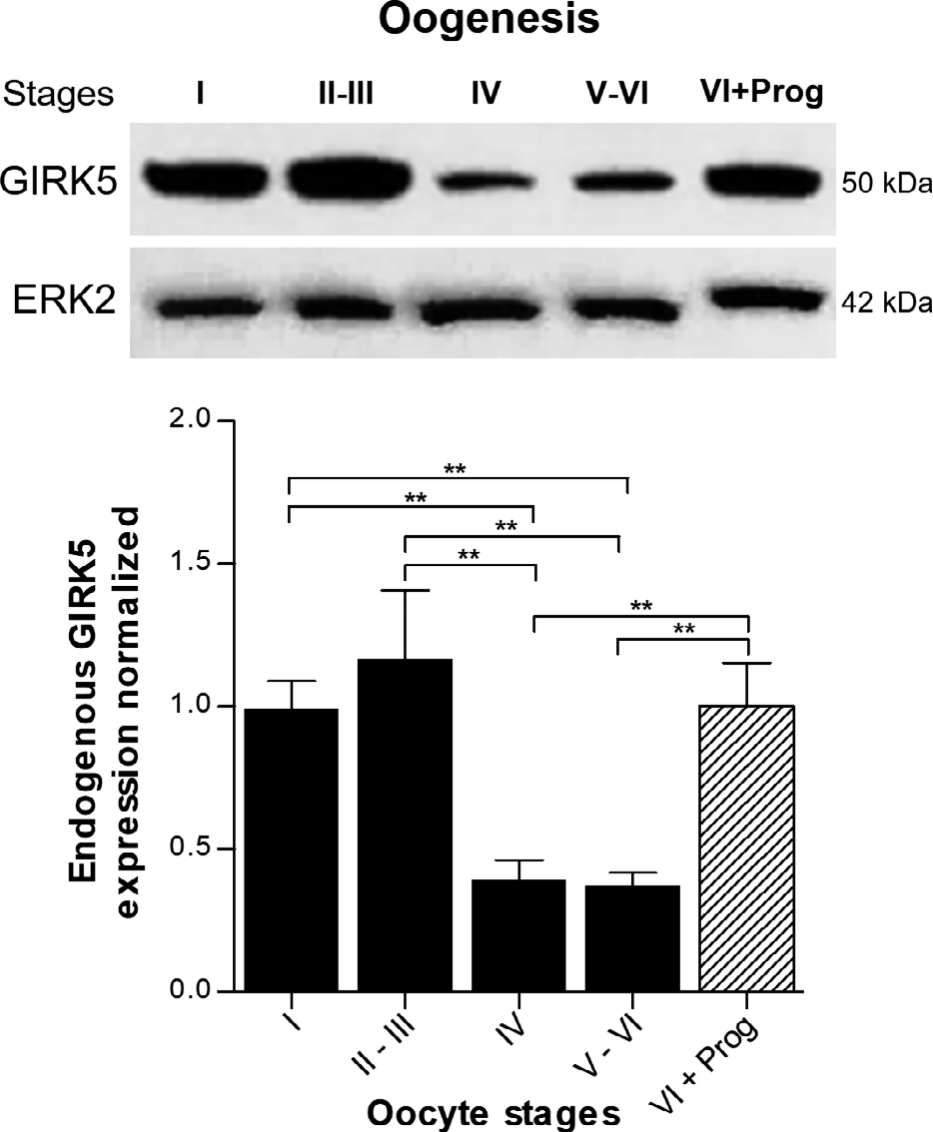
Relative abundance of GIRK5 channel in different stages of *X. laevis* oocytes. GIRK5 expression was evaluated by immunoblotting with a specific antibody (see Materials and methods). (**) Significant difference between the oocytes stages (one‐way ANOVA *P* < 0.05). Error bars correspond to mean ± SE (*n* = 3). VI + Prog, stage VI oocytes incubated with 10 μm progesterone (7 h).

Through the production of a specific antibody, we could determine that endogenous GIRK5 protein level was abundant in early *X. laevis* immature oocytes stages (I–III) but diminished during late stages (IV–VI; Fig. 6). Interestingly, progesterone increased endogenous GIRK5 expression in mature oocytes.

## Discussion

In this work, we found that disruption of the KRXY sequence at the N terminus of GIRK5 is sufficient to change the subcellular location pattern of the channel in the oocyte: from mostly retained in the nucleus, perinuclear space, and the ER to the plasma membrane. These results support that the KRXY sequence corresponds to a non canonical lysine‐arginine‐tyrosine ER retention motif. Furthermore, substitution of lysine 13 for an arginine also disrupted the ER motif (Fig. 1B). Accordingly, if lysine (K) is replaced by an alanine (A) or arginine (R) in the KRR signal of TASK channels (MARRSS and MRRRSS, respectively), protein retention is completely abolished [43].

In particular, membrane proteins are retained in the ER by three types of motifs: KDEL, di‐lysine, and di‐arginine. The KDEL and di‐lysine motifs are retrieval signals that recycle proteins from the Golgi apparatus back to ER; in contrast, the di‐arginine motif avoids proteins to exit the ER. Also, specific signals and position are necessary to retain proteins within the ER [44, 45]. For example, KDEL and di‐lysine motifs need to be located at the C‐terminal of a protein to be recognized as a retrieval signal; in contrast, the di‐arginine motif (RR) is effective, independently of its position [46].

The arginine‐based ER retention/retrieval signal has been well characterized [31, 32, 43]. In the type II membrane proteins there are double arginine motifs (RRXX, RXRXX, XXRR, XRXR) at the N terminus [32]. Lysine residues can replace one by one arginine residue in the RR motif (KR or RK). For example, the two‐pore‐domain K^+^ channels, TASK‐1 and TASK‐3 possess at the N terminus a dibasic KR motif that works as an ER retention motif [43]; but when the two basic residues are separated (KXR), retention of the protein is lost [32].

Previously, we demonstrated that Y16 in GIRK5 could be phosphorylated [30]. Few studies exist about the role of phosphorylation of serine, threonine, and/or tyrosine residues adjacent to ER signal motifs. For example, phosphorylation of PKA and PKC sites flanking the RXR motif in the NR1 subunit of the NMDA receptor suppresses its ER retention [47]. Also, phosphorylation of serine residues adjacent to a retention motif liberates other ion channels and receptors from the ER [48, 49]. Several examples of forward transport of proteins from the ER to the plasma membrane have been documented. Some studies suggest that 14‐3‐3 proteins mediate ER export by interfering with dibasic motifs‐mediated retention [48, 50, 51]. For example, KCNK3 potassium channels contain in the C‐terminal a RRSSV retention motif. KCNK3 binds to 14‐3‐3 protein in a phosphorylation‐dependent manner that suppresses β‐COP binding, allowing forward transport [48]. However, in a preliminary proteomic assay of GIRK5 in oocytes made in our laboratory, 14‐3‐3 protein did not appear to participate (not shown).

The resting membrane potential of fully grown oocytes ranges between −30 and −60 mV in standard saline solution, varies during seasons, and is less negative in mature egg cells [19]. Many attempts have been made to understand the biological significance of ion channels in egg cells. Outward sodium currents have been found only at long‐lasting membrane depolarization in *Xenopus* [52] and *R. pipiens* [53]. However, it is very unlikely that under physiological conditions these sodium channels could be open [54]. Several ionic currents have been detected in native defolliculated *Xenopus* oocytes. These include calcium‐dependent chloride currents [55], voltage‐dependent‐calcium currents [56], voltage‐dependent outward‐rectifying K^+^ currents [57], and stretch‐activated currents [58].

Oocytes are arrested in prophase I of meiosis I. In our study, surface expression of GIRK5 mutants promoted oocyte maturation. In contrast, silencing the expression of GIRK5 or barium block of this channel avoided the maturation process induced by progesterone.

Progesterone oocyte maturation is carried out by the xPR‐1 progesterone membrane receptor [59]. A rapid transient decrease in intracellular cAMP level should happen to release meiotic arrest and then oocyte maturation [60]. The mechanism underlying cAMP decrease involves inhibition of adenylyl cyclase (AC) and/or activation of phosphodiesterases [61]. Free Gβγ subunits of G proteins coupled to AC are natural inhibitors of meiosis in oocytes [14]. In fact, activation of AC maintains high levels of cAMP and meiotic arrest. The *Xenopus* oocyte contains xAC7, an AC activated by Gβγ [62]. Since GIRK5, retained in the ER, helps to maintain oocytes arrested in prophase I of meiosis I, dephosphorylation of tyrosine 16 at the N termini by an endogenous phosphatase would provoke GIRK5 surface expression and oocyte maturation, most probably by binding of Gβγ proteins [63]. Also, GIRK5 might help to maintain prolonged depolarization of the membrane potential necessary to block polyspermy [23]. Further studies should be performed to clarify how surface expression of GIRK5 impacts on oocyte maturation.

Our experimental results also support that *Xenopus* large GIRK5 isoform, containing the KRLY retention motif, is the expressed channel. This finding is probably related to the fact that introns enhance expression levels of their host genes [64]; further experiments are needed to rule out the expression of other GIRK5 isoforms. Interestingly, this large isoform of GIRK5 containing an ER motif was generated because *Xenopus kcnj5* gene acquired two small novel translated exons located upstream from *Kcnj1* gene, converting it into a nested gene. Assis *et al*. [65] reported that animal genomes currently contain several hundreds of nested gene pairs. They found that nearly all nested gene structures in vertebrates seem to have emerged by insertion of a DNA sequence, which arose by gene duplication or retrotransposition, into an intron of a pre‐existing gene [65]. However, in this case, the origin of *Xenopus* nested *kcnj1/kcnj5* genes was due to the acquisition of two novel exons. This last mechanism for the formation of nested gene structures is not unexpected since differences in both sequence and length between orthologs occur predominantly in the NH2 and COOH termini, and probably more examples will be reported. Finally, since these two novel expressed exons are observed only in *X. laevis Kcnj5.L* and *X*. *tropicalis Kcnj5* but not in other tetrapods, including other amphibian relatives, we can conclude that these exons containing the KRLY retention motif were acquired recently in the evolutionary history of *Xenopus*.

High abundance of endogenous GIRK5 and its stage‐dependent level expression during oogenesis is intriguing. Stage VI *Xenopus* oocytes in response to progesterone undergo several biochemical changes, and one of them is an increase in protein synthesis, that will be used later for progression of prophase I‐arrested oocytes through meiosis I [66]. *Xenopus* maternal mRNAs are translationally regulated by cytoplasmic polyadenylation during progesterone‐stimulated oocyte maturation. These maternal mRNAs are not translated *en masse* at any one stage, but instead are activated at specific times in the developing organism. In vertebrates such as frogs (*Xenopus*) and mice, mRNAs encoding the key proteins Mos, Cdk2, and cyclin B are translated during oocyte maturation and regulate such processes as release from meiotic arrest at the end of prophase I, meiotic arrest at metaphase II and mitosis in the embryo. Therefore, our results open new questions related to GIRK5 oocyte stage‐dependent level expression.

In conclusion, we identified a unique lysine‐arginine‐tyrosine retention motif present only in *X. laevis and X. tropicalis*. This retention motif regulates surface expression of GIRK5 and then, maturation of *X. laevis* oocyte. These findings contribute importantly to the knowledge of the developmental biology of *Xenopus*.

## Conflict of interest

The authors declare no conflict of interest.

## Author contributions

LIE conceived and designed the project; CIRG, CS, KCG, BDB, ZLG, LVC, JAVM, VON, and HRR acquired the data; CIRG, KCG, ZLG, and HRR analyzed and interpreted the data; LIE and HRR wrote the paper.

## Supporting information


**Table S1.** GIRK5 primers.

**Data S1.** 7348 protein sequences identified as Kir channels found in 561 completely sequenced animal genomes are listed in fasta format.

## Data Availability

The data that support the findings of this study are available in the [Link], [Link] of this article.
