# Supplementary material for: Identification of a unique endoplasmic retention motif in the Xenopus GIRK5 channel and its contribution to oocyte maturation
Source: FEBS Open Bio. 2021 Mar 3;11(4):1093–108. doi: 10.1002/2211-5463.13113 (PMC8016131; doi:10.1002/2211-5463.13113)
Supplement: Supplementary file 1 — Table S1. GIRK5 primers. [file FEB4-11-1093-s002.docx]

Table S1. Primers used for site-specific mutagenesis.

| **PRIMER** |  | **SEQUENCE** |
| --- | --- | --- |
| K13A |  | Sense  5’ CCTCAGTGGGCAAGATTGTATG 3’  Antisense  5’ CATACAATCTTGCCCACTGAGG 3’ |
| R14A |  | Sense  5’ CAGTGGAAAGCATTGTATGAG 3’  Antisense  5’ CTCATACAATGCTTTCCACTG 3’ |
| K13AR14A |  | Sense |
|  |  | 5’ CCTCAGTGGGCAGCATTGTATG 3’ |
|  |  | Antisense |
|  |  | 5’ CATACAATGCTGCCCACTGAGG 3’ |
| K13AY16A |  | Sense |
|  |  | 5′ CCTCAGTGGGCAAGATTGGCTGAGTCACC 3′ |
|  |  | Antisense |
|  |  | 5′ GGTGACTCAGCCAATCTTGCCCACTGAGG 3′ |
| R14AY16A |  | Sense |
|  |  | 5′ CCTCAGTGGAAAGCATTGGCTGAGTCACC 3′ |
|  |  | Antisense |
|  |  | 5′ GGTGACTCAGCCAATGCTTTCCACTGAGG 3′ |
| K13R |  | Sense |
|  |  | 5’ CCTCAGTGGAGAAGATTGTATG 3’ |
|  |  | Antisense |
|  |  | 5’ CATACAATCTTCTCCACTGAGG 3’ |
| SP6 |  | Sense |
|  |  | 5' GATTTAGGTGACACTATAGAA 3' |
| Low 2 |  | Antisense |
|  |  | 5’ AGAGACCAAAAAGAGACGATCGTCGCCTGTATCAAAG 3′ |
| siRNA 21-mer oligo |  | Top strand |
|  |  | 5‘ CCAAAGAGUUGAAGGAAAUUU 3′ |
